# Lymphangiography with lipiodol as a diagnostic and therapeutic approach for Chyle Leak ascites following Simultaneous Pancreas-Kidney Transplant

**DOI:** 10.1093/jscr/rjab029

**Published:** 2021-03-13

**Authors:** Atta Nawabi, Adam C Kahle, Perel Baral, Clay D King, Perwaiz Nawabi

**Affiliations:** The University of Kansas, Department of Surgery, 3901 Rainbow Blvd, M/S 2005, Kansas City, KS 66160, USA; The University of Kansas, Department of Surgery, 3901 Rainbow Blvd, M/S 2005, Kansas City, KS 66160, USA; The University of Kansas, Department of Surgery, 3901 Rainbow Blvd, M/S 2005, Kansas City, KS 66160, USA; Kansas City University, Kansas City, MO 64106, USA; Kansas City University, Kansas City, MO 64106, USA

## Abstract

Chylous ascites (CA) is the leakage of triglyceride-rich fluid into the peritoneal cavity. This most commonly occurs due to trauma of the lymphatic system. Recently, lymphangiography with lipiodol have been used with promising results in managing refractory postoperative CA. We present the case of a 35-year-old man who developed massive refractory CA post simultaneous pancreas-kidney (SPK) transplant. After conservative management with diet modifications failed, the patient underwent lymphangiography and lymph angioembolization using lipiodol. In this case report, we describe the use of lymphangiography as both a diagnostic and therapeutic approach to successfully manage large volume CA following SPK.

## INTRODUCTION

Simultaneous Pancreas-Kidney (SPK) transplant has become a widely accepted procedure worldwide to achieve glycemic control in patients with Type 1 Diabetes and end-stage renal disease (ESRD). From December 1966 to December 2014 more than 48,000 pancreas transplants worldwide were reported to the International Pancreases Transplant Registry (IPTR), including more than 29,000 from the USA and more than 19,000 outside the US. Post-operative complications are more prevalent when compared to renal transplantation alone. The most common complications cited include metabolic derangements and infection [1], rejection and duodenal leaks [1–5], gastrointestinal bleeding, and malignancy [1].

Lymphocele formation and chylous ascites (CA) are also well-known complications [1]. However, the management of CA is quite varied. Conservative methods include diet modification, total parenteral nutrition (TPN), and the use of pharmacological agents. The use of more invasive procedures like recurrent paracenteses, shunting, embolization, and surgical ligation have also been attempted.

In this case report, we describe the use of lymphangiography as both a diagnostic and therapeutic approach for the management of large volume refractory CA following SPK.

## PRESENTATION OF CASE

A 35-year-old African American male with a history of ESRD secondary to Type 1 diabetes mellitus underwent an SPK transplant with systemic enteric drainage. The anatomy of each organ was normal, and the procedure went very well. On postoperative day (POD) 4, the peri-pancreatic Jackson-Pratt drain was noted to have a cloudy white appearance. Fluid from the drain was tested and revealed a triglyceride level of 501 mg/dL, nearly five times over his serum triglyceride level of 91 mg/dL. Prompt diagnosis of a Chyle leak was made and the patient was subsequently started on chyle diet consisting of a high-protein, low-fat diet with medium-chain triglycerides. Examination of drain output On POD 9 revealed serous fluid prompting removal of the drain. The patient was discharged home afterword.

**Figure 1:**
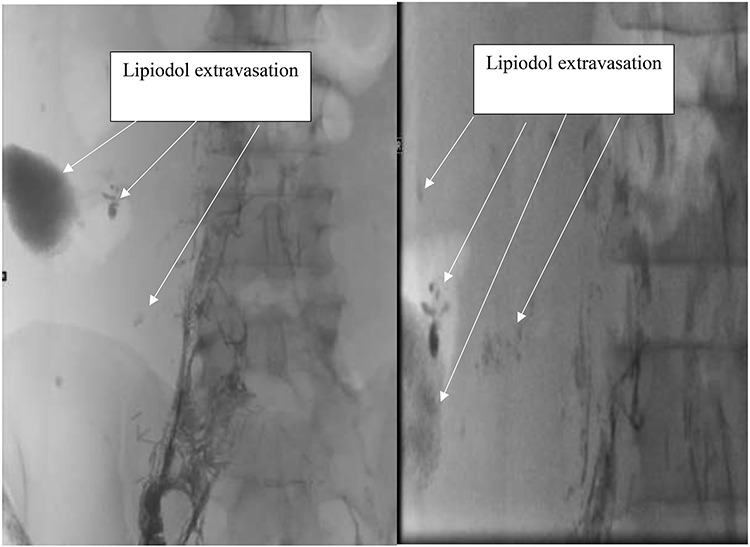
Lymphangiogram with lipiodol showing Lipiodol extravasation at the site of chyle leak.

Follow up with the patient occurred one week later revealed recurrent chyle ascites. Failure of conservative management with the chyle diet prompted consultation with Interventional radiology for lymphangiography and possible lymph angioembolization. Imaging from lymphangiography with Lipiodol demonstrated pooling of contrast and lipiodol in the right lower quadrant, which favored the chyle leak site seen in Fig. 1. Two days later after lymphangiogram with lipiodol the leak was ceased, and the patient was discharged home.

## DISCUSSION

Chyle ascites is the extravasation of triglyceride-rich fluid in the abdominal cavity [4]. It is diagnosed by a fluid triglyceride level of >200 mg/dL. The overall incidence of CA reported after abdominal surgery is <1%. Surgeries that involve extensive mobilization and dissection in the retroperitoneum and pelvis have higher incident rates of 4.7% to 7.4% [5–7].

The initial treatment for chyle ascites includes the introduction of a high-protein, low-fat diet with medium-chain triglycerides. Following diet modification failure, total bowel rest and TPN are initiated to slow intestinal lymphatic flow [8]. Other measures, such as the use of somatostatin analogs, porto-venous shunting, and trans jugular intrahepatic portosystemic shunting have been utilized to reduce lymphatic hypertension and return chylous drainage into blood circulation [8]. Surgical ligation is also a treatment option for refractory CA after failed conservative treatment; however, surgery is invasive with the potential failure to locate a leak.

Chyle leak after simultaneous kidney-pancreas transplant is not an uncommon complication given the amount of dissection needed for pancreas transplantation. Special attention should be given when dissecting to ensure ligation of lymphatic channels. Currently, the management of Chyle leaks has been with drains, a low-fat diet, and electrolyte replenishment [3]. We demonstrate in our case the recurrence of chyle ascites following conservative management. Effective and definitive treatment of this complication can be achieved through Interventional Radiology utilizing lymphangiography and lymph angioembolization to localize and treat the chyle leak. Use of lymphangiography has been reported for treatment of intrathoracic chyle leaks after intrathoracic surgery [9, 10] but to the best of our knowledge, we report the first case of its use in a chyle leak following SPK.

Treatment options for CA range from conservative strategies such as diet modification and pharmacologic therapies, to more invasive procedures like surgical ligation of lymphatic channels. A common approach is to initiate a high-protein, low-fat diet with medium-chain triglycerides. Following diet modification failure, total bowel rest and TPN are initiated to avoid intestinal lymphatic flow [8]. Other measures, such as the use of somatostatin, Porto venous shunting, and trans jugular intrahepatic portosystemic shunting can relieve the ascites to reduce lymphatic hypertension and return chylous drainage into blood circulation [8]. Surgical ligation is also a treatment option for refractory CA after failed conservative treatment; however, surgery is invasive and may fail to locate the leak.

Lymphangiography may be used as a diagnostic and potential therapeutic approach for CA. Lymph angioembolization can also be utilized concurrently. Furthermore, lymphangiography alone has been shown to dramatically slow down or completely stop the chyle leaks in our cases. This is believed to be due to lipiodol accumulating at the point of leakage resulting in a regional inflammatory reaction occurring in the soft tissues adjacent to the lipiodol retention yielding embolization [11]. The viscosity of lipiodol is also thought to contribute to embolization.

To our knowledge, this case report highlights the first use of lymphangiography with lipiodol as a successful diagnostic and therapeutic approach to CA after SPK. Further follow up needed to assess long-term outcome of the patient.
